# Docosahexaenoic acid inhibits TNF-α-induced osteoclast formation and orthodontic tooth movement through GPR120

**DOI:** 10.3389/fimmu.2022.929690

**Published:** 2023-01-18

**Authors:** Jinghan Ma, Hideki Kitaura, Saika Ogawa, Fumitoshi Ohori, Takahiro Noguchi, Aseel Marahleh, Yasuhiko Nara, Adya Pramusita, Ria Kinjo, Kayoko Kanou, Akiko Kishikawa, Atsuhiko Ichimura, Itaru Mizoguchi

**Affiliations:** ^1^ Division of Orthodontics and Dentofacial Orthopedics, Tohoku University Graduate School of Dentistry, Sendai, Miyagi, Japan; ^2^ Frontier Research Institute for Interdisciplinary Sciences, Tohoku University, Sendai, Miyagi, Japan; ^3^ Department of Biological Chemistry Graduate School of Pharmaceutical Sciences, Kyoto University, Kyoto, Japan

**Keywords:** osteoclast, TNF-α, GPR120, DHA, orthodontic tooth movement

## Abstract

Docosahexaenoic acid (DHA) is an omega-3 fatty acid that has a range of positive impacts on human health, including anti-inflammatory effects and inhibition of osteoclast formation *via* G-protein-coupled receptor 120 (GPR120). Orthodontic force was reported to induce tumor necrosis factor-α (TNF-α) expression, which activates osteoclast differentiation during orthodontic tooth movement (OTM). The aim of this study was to investigate the influence of DHA on TNF-α-induced osteoclast formation and OTM *in vivo*. We examined osteoclast formation and bone resorption within the calvaria of both wild-type (WT) and GPR120-deficient (GPR120-KO) mice injected with phosphate-buffered saline (PBS), TNF-α, TNF-α and DHA, or DHA. DHA inhibited TNF-α-induced osteoclast formation and bone resorption in WT mice but had no effect in GPR120-KO mice. OTM experiments were performed in mouse strains with or without regular injection of DHA, and the effects of DHA on osteoclast formation in the alveolar bones during OTM were examined. DHA also suppressed OTM in WT but not GPR120-KO mice. Our data showed that DHA suppresses TNF-α-induced osteoclastogenesis and bone resorption *via* GPR120. TNF-α has considerable significance in OTM, and therefore, DHA may also inhibit TNF-α-induced osteoclast formation and bone resorption in OTM.

## Introduction

Docosahexaenoic acid (DHA), a long-chain n-3 polyunsaturated fatty acid with 22 carbon atoms and 6 double bonds, is one of the polyunsaturated fatty acids. It has been shown to have benefits on several disorders, including cardiovascular disease (1), diabetes (2), inflammatory bowel disease, rheumatoid arthritis, dysmenorrhea, asthma, atherosclerosis, and autoimmune diseases (3–6). Moreover, supplements containing DHA have shown positive effects on some cancers, diabetes, and cardiovascular diseases (7, 8).

Osteoclasts, the derivative of the hematopoietic stem cells with multi-nucleus, are prerequisites for bone remodeling through destruction of the bone matrix and bone erosion such as rheumatoid arthritis, periprosthetic bone loss, periodontal disease, and postmenopausal osteoporosis (9). The differentiation and functional maturation of osteoclasts are mediated by two cytokines. One is the receptor activator of NF-kB ligand (RANKL), and the other is the macrophage colony-stimulating factor (M-CSF) (10). Additionally, tumor necrosis factor-α (TNF-α) promotes the differentiation of osteoclasts from osteoclast precursors derived from bone marrow cells both *in vitro* (11–13) and *in vivo* (14, 15). TNF-α has significant functions during osteoerosive disorders, including inflammation (16).

The G-protein-coupled receptors (GPRs) are seven transmembrane domain receptors that play crucial roles in many cellular functions and in the regulation of several physiological and pathological processes (17–20). Long-chain fatty acids bind the free fatty acid receptors (FFARs), GPR120 (FFAR4), GPR41 (FFAR3), GPR43 (FFAR2), GPR40 (FFAR1), and GPR84 (21, 22), among which GPR120 plays a key role in homeostatic metabolic regulation during immune processes and inflammation *in vivo* (23). Thus, the GPRs have attracted a great deal of attention as major drug development targets for several human diseases, and research has focused on GPR120 due to its role in inflammatory diseases, including obesity and diabetes (17, 22–27).

Dietary n-3 fatty acids prevent bone destruction in ovariectomized mice, and n-3, but not n-6, fatty acids inhibit osteoclast formation *in vitro* (28). Several *in vitro* studies showed that DHA has a suppressive effect on osteoclast formation and function and on the differentiation of human CD14^+^ monocytes into osteoclasts (29). DHA has also restrictive effects on osteoclast differentiation, activation, and function of the mouse monocyte cell line RAW 264.7 mediated *via* stimulation of RANKL *in vitro* (30). Our previous *in vitro* study showed that DHA attenuated osteoclast differentiation induced by both RANKL and TNF-α (31). Furthermore, DHA attenuates lipopolysaccharide (LPS)-induced osteoclast formation and bone resorption *in vivo* (31). These suppressive effects of DHA are mediated by the inhibition of TNF-α expression induced by LPS in macrophages and by inhibition of both osteoclast formation induced by RANKL and by TNF-α directly through GPR120 (31).

GPR120 signaling inhibits osteoclast formation by suppressing RANKL-mediated nuclear factor-kappa B (NF-kB) activation and nuclear factor of activated T-cell cytoplasmic (NFATc1) induction (32). Moreover, FFAs, including DHA, prevent the nuclear translocation of NF-κB in murine macrophages; however, the translocation of NF-κB subunit to the nuclei was not blocked in the case of FFAR4 absence (33). According to our previous study, NF-κB is involved in TNF-α-induced transcriptional activation of inflammation-associated genes. However, the effect of DHA on TNF-α-induced NF-κB pathway activation through GPR120 is still unclear.

Orthodontic tooth movement (OTM) with the application of orthodontic force involves the remodeling of alveolar bone and reconstruction of the periodontal ligament. Multiple factors are involved in the mechanism underlying OTM, including cytokines, growth factors, components of the bone matrix, and neurotransmitters, which mediate the differentiation and function of osteoblasts and osteoclasts, causing bone remodeling (34–38).

Several studies have demonstrated that mechanical force loading induced the production of TNF-α (39, 40). Moreover, TNF-α receptor-deficient mice reduced tooth movement and osteoclast formation compared to wild-type (WT) controls, which suggested that TNF-α has remarkable effects on osteoclast formation and bone resorption in OTM (41, 42). Our earlier research has found that DHA directly inhibits TNF-α-induced osteoclast formation, suggesting that DHA may also inhibit the TNF-α-induced osteoclastogenesis and bone destruction in OTM (31). However, the effects of DHA on OTM are yet to be elucidated.

The present research was performed with the aim of evaluating the effects of DHA injection on TNF-α-induced osteoclastogenesis and bone destruction in mice through GPR120, and elucidating the underlying mechanisms by *in vitro* experiments. Furthermore, we examined the influence of DHA on OTM and the levels of osteoclast activity mediated *via* GPR120.

## Materials and methods

### Animals and reagents

Male C57BL6/J mice, 8–12 weeks old, were purchased from CLEA Japan (Tokyo, Japan) to be used as WT mice. GPR120-KO mice were generated by crossing mice expressing Cre-recombinant activity under the influence of chicken actin promoter and C57BL6/J mice bearing Ffar4-flowed gene (Ffar4^(dE1/dE1)^ mice) (31). All animal care and experiments were approved by the Tohoku University of Science Animal Care and Use Committee (2020DnA-007-05). DHA was obtained from Sigma-Aldrich (St. Louis, MO, USA). Recombinant murine TNF-α was obtained as described previously (14), and recombinant mouse M-CSF was obtained from CMG14-12, M-CSF-expressing cell line (43).

### Histological analysis

WT and GPR120-KO mice were divided into four groups (*n* = 4), which received daily subcutaneous supracalvarial injection of the following for 5 consecutive days: negative control, phosphate-buffered saline (PBS); TNF-α (3 μg/day); DHA (100 µg/day) and TNF-α (3 μg/day); and DHA (100 µg/day). On day 6, all mice were sacrificed, and the calvariae were resected and then fixed immediately in 4% PBS-buffered formaldehyde. After fixation for 24 h at 4°C, 14% ethylenediaminetetraacetic acid (EDTA) was used to demineralize all calvariae for 3 days at room temperature. After dehydration in a tissue processer (TP1020; Leica Wetzlar, Germany), all calvariae were embedded using paraffin and then cut into 5-µm sections perpendicular to the sagittal suture using a microtome. Paraffin sections were stained with TRAP solution mixed with acetate buffer (pH 5.0), naphthol AS-MX phosphate (Sigma-Aldrich), Fast Red Violet LB Salt (Sigma-Aldrich), and 50 mM sodium tartrate. After staining, all sections were counterstained with hematoxylin. Cells were considered as osteoclasts if they were TRAP-positive and contain three or more nuclei (40). TRAP-positive osteoclasts at the suture mesenchyme of mice sagittal suture were counted and normalized to 400 × 400 µm^2^ surface area of each section.

To analyze OTM, the maxillae were fixed in 4% formaldehyde in PBS before being decalcified with 14% EDTA. Samples were dehydrated in a tissue processer (TP1020; Leica), before being embedded in paraffin. The upper left first molars of these mice were cut into horizontal sections at 100, 140, 180, 220, and 260 μm from the apical of the bifurcation area. All the sections were stained with TRAP solution and counterstained with hematoxylin. The mesial side of the labiolingual long axis of the distobuccal root was regarded as the compression side. Finally, we measured the number of osteoclasts on the alveolar surface of the compression side on the sections of different levels to analyze osteoclast formation (44).

### Micro-CT analysis of bone destruction area

Mice treated with daily subcutaneous supracalvarial injection were sacrificed on day 6. Their calvariae were fixed immediately in 4% PBS-buffered formaldehyde and then scanned using microfocus computed tomography (micro-CT) (ScanXmate-E090; Comscan, Kanagawa, Japan). Software TRI/3DBON64 (RATOC System Engineering, Tokyo, Japan) was used to create 3D images of the calvariae. The resorption area was regarded as the black area between the sutures, in contrast to the bone surface, which appears white in the 3D CT images. To quantify the bone resorption area, a 50 × 70 pixel rectangle was selected at the intersection between the sagittal and coronal sutures, and the amount of bone resorption area to the total area was measured using ImageJ software (NIH, Bethesda, MD, USA) (40).

### Preparation of osteoblasts

The calvariae of 5- to 6-day-old WT and GPR120-KO mice were dissected. Collagenase solution (0.2% w/v; Wako Pure Chemical Industries, Osaka, Japan) was prepared in isolation buffer (3 mM K_2_HPO_4_, 10 mM NaHCO_3_, 60 mM sorbitol, 70 mM NaCl, 1 mM CaCl_2_, 0.5% [w/v] glucose, 0.1% (w/v) bovine serum albumin [BSA], and 25 mM 4-(2-hydroxyethyl)-1-piperazineethanesulfonic acid). EDTA was prepared at a concentration of 5 mM with 0.1% BSA in PBS and filtered through a 0.2-µm filter. Calvariae were subjected to digestion *via* collagenase and EDTA, for 20 and 15 min, respectively, at 37°C on a shaker. Fraction 1 (collagenase), fraction 2 (EDTA), fraction 3 (collagenase), fraction 4 (collagenase), fraction 5 (EDTA), and the digests were collected. Fractions 3–5 were considered as the osteoblast high fractions. Cells were cultured in alpha minimal essential medium (α-MEM) (Wako), containing 10% fetal bovine serum (FBS) and 100 IU/ml penicillin G and 100 μg/ml streptomycin, overnight. The adherent cells were collected using trypsin-EDTA (Life Technologies, Grand Island, NY, USA). The cells were cultured in α-MEM containing 10% FBS for 3 days with the culture media changed every 2 days. Adherent cells were used as osteoblasts (45).

### RNA preparation and real-time RT-PCR analysis

The calvariae of mice from all eight groups of the *in vivo* experiments were frozen in liquid nitrogen and then crushed using Micro Smash MS-100R (Tomy Seiko, Tokyo, Japan) in 800 μl of TRIzol reagent (Invitrogen, Carlsbad, CA, USA). RNA was also prepared from osteoblasts of the mouse strains from the *in vitro* experiments. Cells were incubated in α-MEM with either PBS, TNF-α (100 ng/ml), TNF-α (100 ng/ml) and DHA (100 ng/ml), or DHA (100 ng/ml) for 3 days. Total RNA was used to synthesize cDNAs of each group of samples with oligo-dT primers (Invitrogen) and reverse transcriptase in a total volume of 20 μl. Each reaction contained 2 μl of cDNA and 23 μl of a mixture consisting of SYBR Premix Ex Taq (Takara, Shiga, Japan) and 50 pmol/μl of primers. TRAP, RANKL, and OPG mRNA expression levels were evaluated using real-time RT-PCR using a Thermal Cycler Dice Real-Time System (Takara). The PCR cycling conditions were started with an initial denaturation step at 95°C for 10 s, followed by 45–60 cycles of amplification, with each cycle consisting of a denaturation step at 95°C for 5 s and an annealing step at 60°C for 30 s. To determine the relative expression levels of RANKL mRNAs, glyceraldehyde 3-phosphate dehydrogenase (GAPDH) was used as a reference gene. The primer sequences used for cDNA amplification were as follows: GAPDH, 5′-GGTGGAGCCAAAAGGGTCA-3′ and 5′-GGGGGCTAAGCAGTTGGT-3′; TRAP, 5′-AACTTGCGACCATTGTTA-3′ and 5′-GGGGACCTTTCGTTGATGT-3′; RANKL, 5′-CCTGAGGCCAGCCATTT-3′ and 5′-CTTGGCCCAGCCTCGAT-3′; OPG, 5′-CTTAGGTCC AACTACAGAGGAAC-3′ and 5′-ATCAGAGCCTCATCACCTT-3′; and Cathepsin K 5′-GCAGAGGTTGTACTATGA-3′ and 5′-GCAGGCGTTGTTCTTATT-3′ (46).

### Preparation of osteoclast precursors and co-culture of both GPR120-KO osteoclast precursors and WT or GPR120-KO osteoblasts

The bone marrow cells of GPR120-KO mice were prepared by flushing from the femora and tibiae and cultured in α-MEM with 10% FBS, 100 IU/ml penicillin G, and 100 μg/ml streptomycin together with 100 ng/ml M-CSF for 3 days. Attached cells were obtained by trypsin-EDTA (Life Technologies). These cells were collected as osteoclast precursors. GPR120-KO osteoclast precursors and WT or GPR120-KO osteoblasts were co-cultured in the presence of 10^−6^ M PGE2+10^−8^ M 1,25(OH)_2_D_3_ (Sigma-Aldrich) with or without TNF-α (100 ng/ml) and with or without DHA in 96-well plates. Co-culturing was continued for 4 days after which it was ended by fixation with 4% formaldehyde and stained with TRAP as described previously (47). Cells were considered as osteoclasts if they were TRAP positive and contained three or more nuclei.

### Immunofluorescence

Osteoblasts isolated from WT or GPR120KO mice were seeded in 96-well plates in α-MEM at a density of 3 × 10³ per well overnight at 37°C. Cells were then stimulated with PBS, TNF-α (100 ng/ml), TNF-α (100 ng/ml) and DHA (100 ng/ml), or DHA (100 ng/ml) for 1 h. After this, we removed the culture medium and washed it three times using PBS. We then fixed it in 4% (w/v) formaldehyde solution in PBS for 15 min at room temperature. The fixative solution in the 96-well plates was removed and washed by PBS before adding 0.5% Triton X-100 (v/v) in PBS for 15 min. All the wells were washed with PBS and blocked with 3% BSA in PBS. Wells were incubated with p65 (D14E12) XP Rabbit mAb (Cell Signaling Technology, MA, USA; 1:400 dilution) diluted in 3% BSA in PBS to 1:400 overnight at 4°C. Cells were washed and incubated with Alexa Fluor 555 conjugate (Cell Signaling Technology, MA, USA; 1:100 dilution) in 3% BSA in PBS for 1 h in the dark at room temperature. Finally, cells were incubated with 4′,6-diamidino-2-phenylindole (DAPI) for 5 min before visualizing fluorescent cells under a fluorescence microscope (Olympus IX71, Tokyo, Japan).

### Preparation of OTM and measurement of the distance of tooth movement

OTM was performed as described previously (48). Twelve-week-old WT or GPR120-KO male mice received injections of PBS or DHA (100 µg/day) every 2 days during OTM using a 30 G 10-mm needle under anesthesia. There was a total of seven injections for each mouse during the 12-day OTM. The injection site was as close to the gingiva on the buccal side as possible, and there was only one injection site at a time. The upper incisor and upper left first molar were bound with a nickel–titanium (Ni–Ti) closed-coil spring (Tomy Seiko) under anesthesia. This appliance was fixed with a stainless-steel wire (0.01 mm in diameter) and attached to a hole through the upper anterior alveolar of the mice to move the upper left first molar mesially with 10*g* tractive force. After 12 days of force loading, the distance between the upper first molar and the second molar was measured with silicone impressions of the maxilla, and the distance was compared between groups. These alveolar bones were also used for histological analysis.

### Statistical analysis

All data were expressed as the mean ± standard deviation (SD). Statistical analyses were performed using Scheffé’s test and t-test. In all analyses, *p* < 0.05 represented statistical significance.

## Results

### DHA inhibited osteoclast formation induced by TNF-α through GPR120 activation *in vivo*

**Figure 1:**
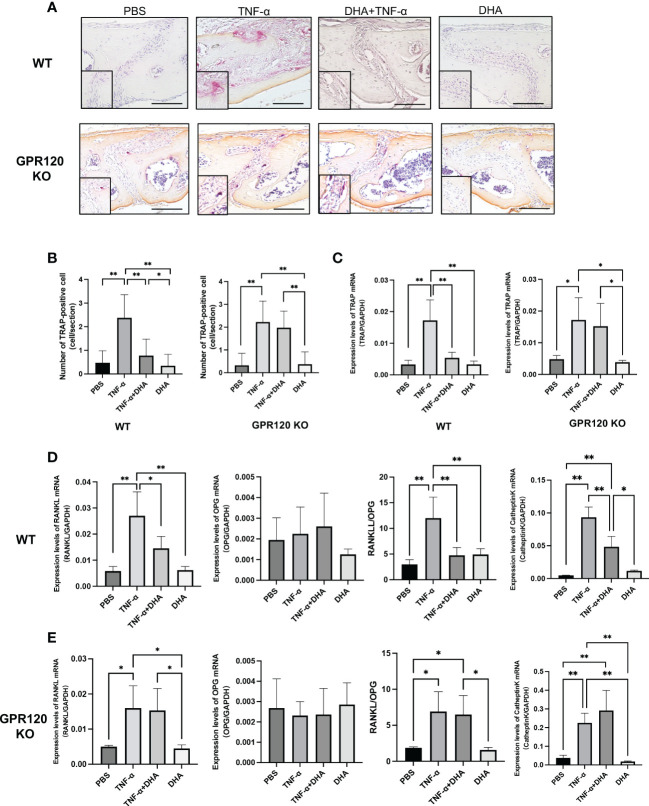
DHA inhibited osteoclast formation induced by TNF-α *via* GPR120 *in vivo.* **(A)** Microscopic images of TRAP-stained sections of mouse calvariae. Calvariae of WT and GPR120-KO mice receiving a 5-day daily subcutaneous injection of PBS, TNF-α, TNF-α + DHA, or DHA only to supracalvaria were cut into 5-µm sections. After staining with TRAP solution, hematoxylin was used as a counterstain for those sections. **(B)** TRAP-positive cells number in the sagittal suture of four groups of WT and GPR120-KO mice administered PBS, TNF-α, TNF-α + DHA, or DHA. **(C)** TRAP mRNA levels in WT and GPR120-KO mouse calvariae were determined using real-time RT-PCR. **(D)** RANKL, OPG, and Cathepsin K mRNA levels and the ratio of RANKL/OPG in WT mouse calvariae were determined using real-time RT-PCR. **(E)** RANKL, OPG, and Cathepsin K mRNA levels and the ratio of RANKL/OPG in GPR120-KO mouse calvariae were determined using real-time RT-PCR. The results are given as means ± SD. Scheffé’s test was used to determine the statistical significance of differences (n = 4; *p < 0.05, **p < 0.01). Scale bars = 100 µm.

We examined whether osteoclast formation induced by TNF-α is inhibited by DHA through GPR120 *in vivo*. TNF-α and DHA were injected into the calvariae of both WT and GPR120-deficient (GPR120-KO) mice. Following 5 consecutive days of TNF-α injection, large numbers of TRAP-positive cells with three or more nuclei were recognized along the suture mesenchyme on histological sections. In contrast, WT mice co-administered TNF-α and DHA showed a significant reduction in the mean number of TRAP-positive cells compared to those treated with TNF-α alone (**Figures 1A, B**). However, the inhibitory effect of DHA was attenuated in GPR120-KO mice (**Figures 1A, B**). Furthermore, TRAP, RANKL, and Cathepsin K mRNA levels were significantly lower in WT mice co-administered TNF-α and DHA than in those administered TNF-α alone (**Figures 1C, D**), so as the ratio of RANKL/OPG. However, there were no significant differences in TRAP, RANKL, or Cathepsin K mRNA levels in GPR120-KO mice administered TNF-α with or without DHA injection (**Figures 1C–E**), and the ratio of RANKL/OPG was also at the same level in these two groups of GPR120 KO mice.

### DHA inhibited bone resorption induced by TNF-α through GPR120 activation *in vivo*

**Figure 2:**
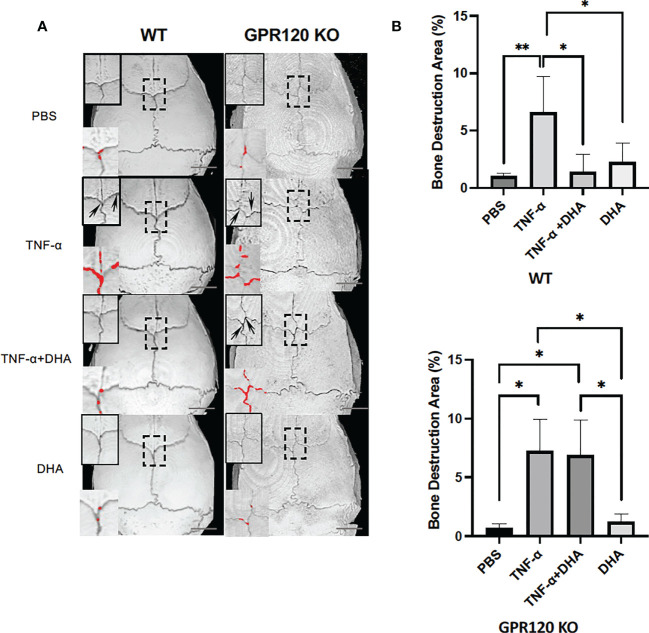
DHA inhibited TNF-α-induced bone resorption *in vivo*. **(A)** Three-dimensional reconstructed images of WT and GPR120 mouse calvariae. After receiving daily injections of PBS, TNF-α, TNF-α + DHA, or DHA only for 5 days, calvariae were resected and scanned using micro-CT. Three-dimensional images were created using TRI/3DBON64 software. Bone destruction areas are indicated by red dots and upper left corner zoom in of each image with arrows. **(B)** The ratio of bone destruction area to total calvarial bone area in WT and GPR120-KO mice. The results are given as means ± SD. Scheffé’s test was used to determine the statistical significance of differences (n = 4; *p < 0.05, **p < 0.01) Scale bars = 2 mm.

After scanning using micro-CT, the ratio of bone resorption area to the total area of mice calvariae was analyzed. Mice injected with TNF-α showed a considerably larger bone resorption area than the PBS control or DHA groups. However, the bone resorption area was markedly decreased in the TNF-α and DHA co-administration group. In contrast, there were no significant differences in bone resorption area in GPR120-KO administered TNF-α with or without DHA (**Figures 2A, B**).

### DHA inhibited TNF-α-induced RANKL expression in osteoblasts through GPR120 activation *in vitro*

**Figure 3:**
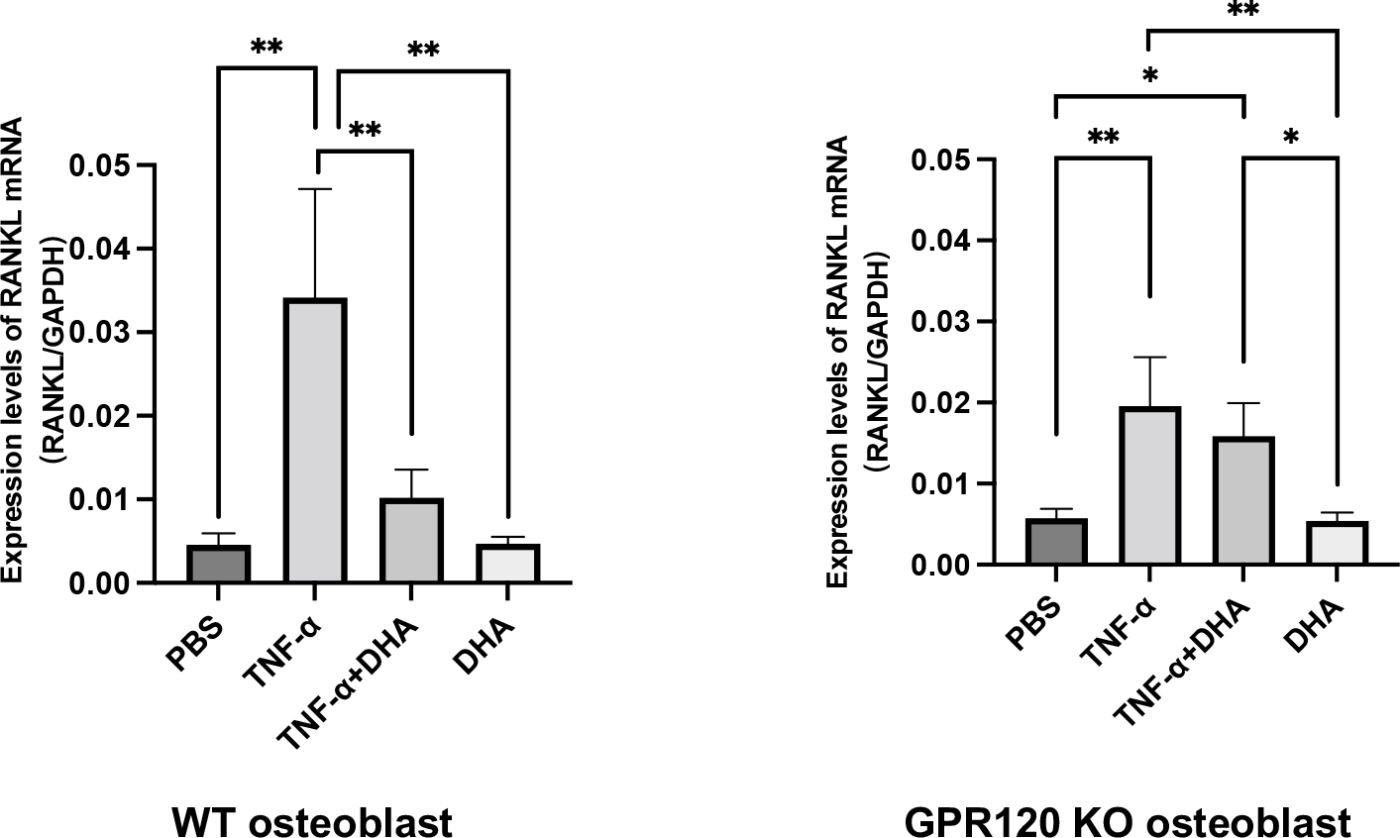
DHA inhibited RANKL expression in osteoblasts *via* GPR120 *in vitro*. RANKL mRNA levels of osteoblasts were demonstrated using real-time RT-PCR. Osteoblasts of WT and GPR120-KO mice were cultured with PBS, TNF-α, TNF-α + DHA, or DHA only. The results are given as means ± SD. Scheffé’s test was used to determine the statistical significance of differences (n = 4; *p < 0.05, **p < 0.01).

We analyzed RANKL mRNA expression levels in osteoblasts *in vitro* to determine the mechanism by which DHA inhibits osteoclast formation through activation of GPR120. RANKL mRNA expression level was higher in the group treated with TNF-α alone than that in the control group (PBS) or the DHA-treated group. In contrast, the group treated with both TNF-α and DHA showed a lower RANKL mRNA expression than the group treated with TNF-α. On the contrary, the expression level of RANKL mRNA was similar in GPR120-KO osteoblasts treated with TNF-α and with TNF-α plus DHA (**Figure 3**).

### DHA inhibited TNF-α-enhanced osteoclast formation in co-culture *via* GPR120 activation in osteoblasts

**Figure 4:**
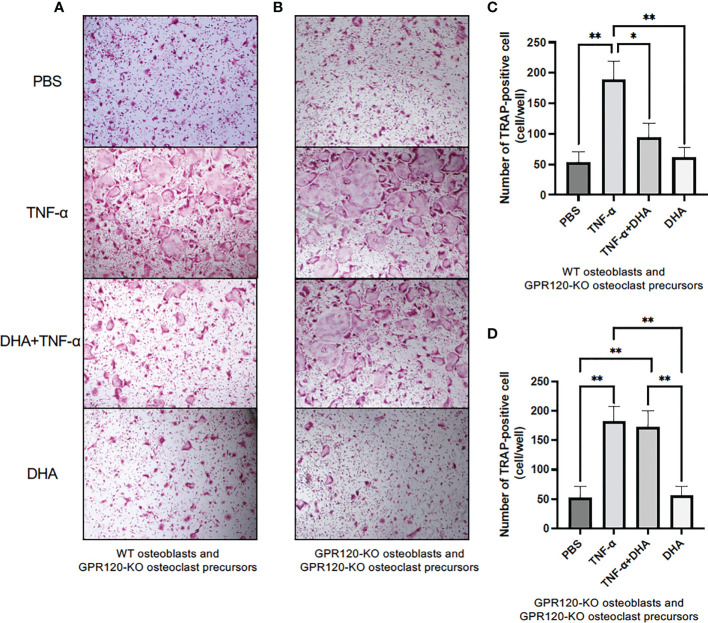
DHA inhibited TNF-α-induced osteoclast formation in co-culture using GPR120-KO osteoclast precursors to avoid the effects of DHA on osteoclastogenesis and WT osteoblasts or GPR120-KO osteoblasts. **(A)** Images of TRAP-positive cells and **(C)** the numbers of TRAP-positive cells in co-cultures of GPR120-KO osteoclast precursors and WT osteoblasts cultured with PBS, TNF-α (100 ng/mL), TNF-α + DHA, or DHA only in the presence of prostaglandin E2 and 1,25(OH)2D3 for 4 days. **(B)** Images of TRAP-positive cells and **(D)** the numbers of TRAP-positive cells in co-cultures of GPR120-KO osteoclast precursors with GPR120-KO osteoblasts cultured with PBS, TNF-α (100 ng/ml), TNF-α + DHA, or DHA only in the presence of prostaglandin E2 and 1,25(OH)2D3 for 4 days. The results are given as means ± SD. Scheffé’s test was used to determine the statistical significance of differences (n = 4; *p < 0.05, **p < 0.01).

To examine whether TNF-α induced RANKL in osteoblasts and enhanced osteoclastogenesis, GPR120-KO osteoclast precursors were used to avoid the direct effects of DHA on differentiation of osteoclast precursors to osteoclasts. Osteoblasts from WT mice or GPR120-KO and GPR120-KO osteoclast precursors were cultured with PBS as a control, TNF-α (100 ng/ml), TNF-α (100 ng/ml) and DHA (100 ng/ml), or DHA (100 ng/ml) in the presence of 10^−8^ M 1,25(OH)_2_D_3_ and 10^−6^ M prostaglandin E2. The numbers of TRAP-positive cells were measured in co-cultures, and these results indicated a significant decrease in the TRAP-positive cell number associated with DHA treatment in co-culture using WT osteoblasts but not GPR120-KO osteoblasts (**Figures 4A–D**).

### DHA inhibited NF-κB activation induced by TNF-α in osteoblast through GPR120

**Figure 5:**
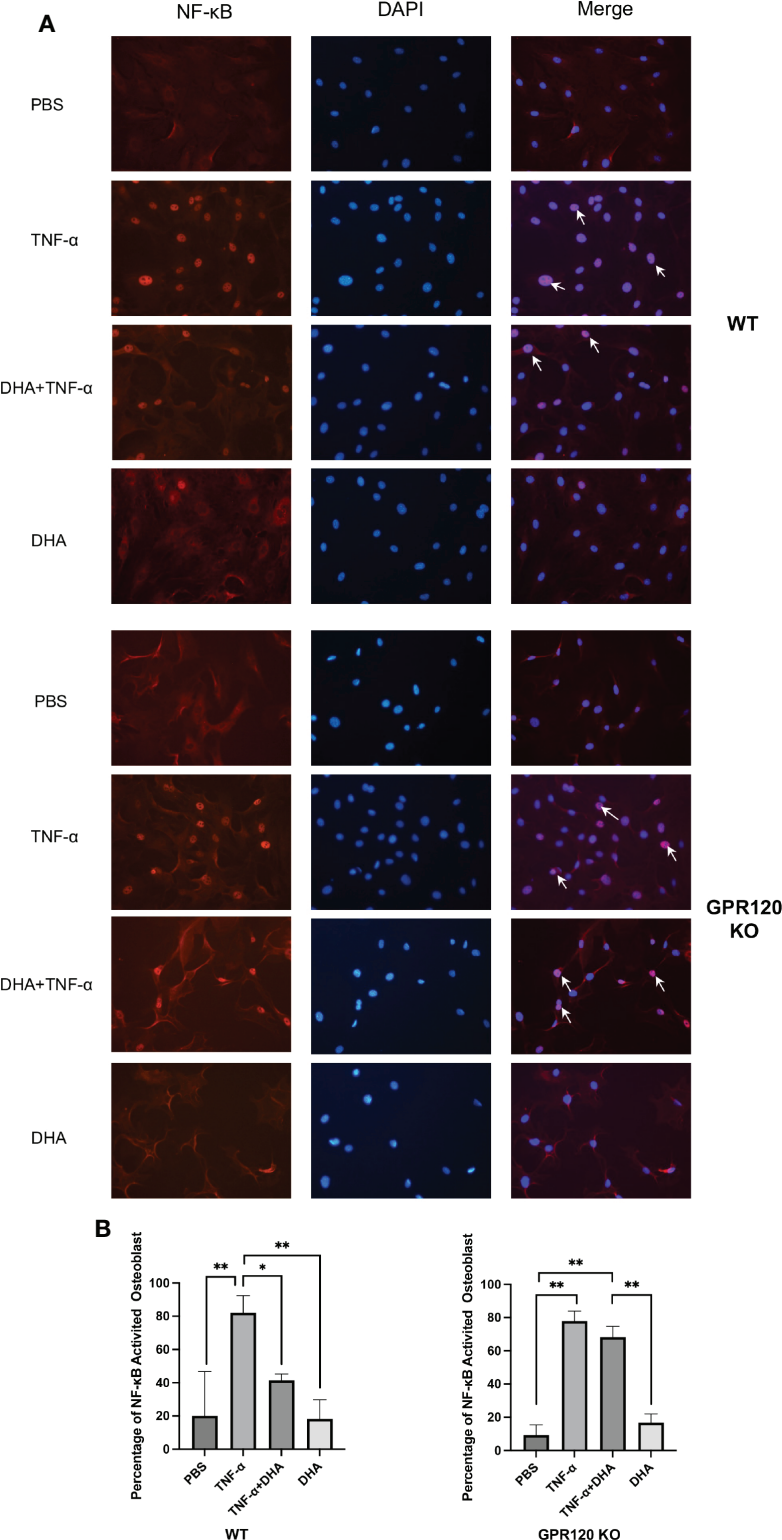
Effect of DHA on NF-κB activation by TNF-α in osteoblast. **(A)** Fluorescent images of WT osteoblast stained for NF-κB p65 antibody and Alexa Flour 555; DAPI was used as control. NF-κB activated osteoblast were indicated by arrows. **(B)** NF-κB activated osteoblasts number to the total number of osteoblasts. The results are given as means ± SD. Scheffé’s test was used to determine the statistical significance of differences (n = 4; *p < 0.05, **p < 0.01). Scale bars = 50 μm.

To investigate the role of DHA in the transcription factor NF-κB pathway, we divided the WT and KO cells into four groups and applied stimulation of PBS as a control, TNF-α (100 ng/ml), TNF-α (100 ng/ml) and DHA (100 ng/ml), or DHA (100 ng/ml). We found that NF-κB nuclear localization significantly increased in WT osteoblasts when osteoblasts received TNF-α stimulation, while it was reduced when DHA and TNF-α were co-administered (**Figures 5A, B**). This suggests that DHA inhibits the NF-κB signaling pathway, which is activated by TNF-α. However, this inhibitory effect was eliminated in GPR120 KO mice, and DHA did not exert an inhibitory effect on the NF-κB pathway activated by TNF-α in the osteoblasts of GPR120-KO mice (**Figures 5A, B**).

### DHA inhibited OTM *via* GPR120 activation

**Figure 6:**
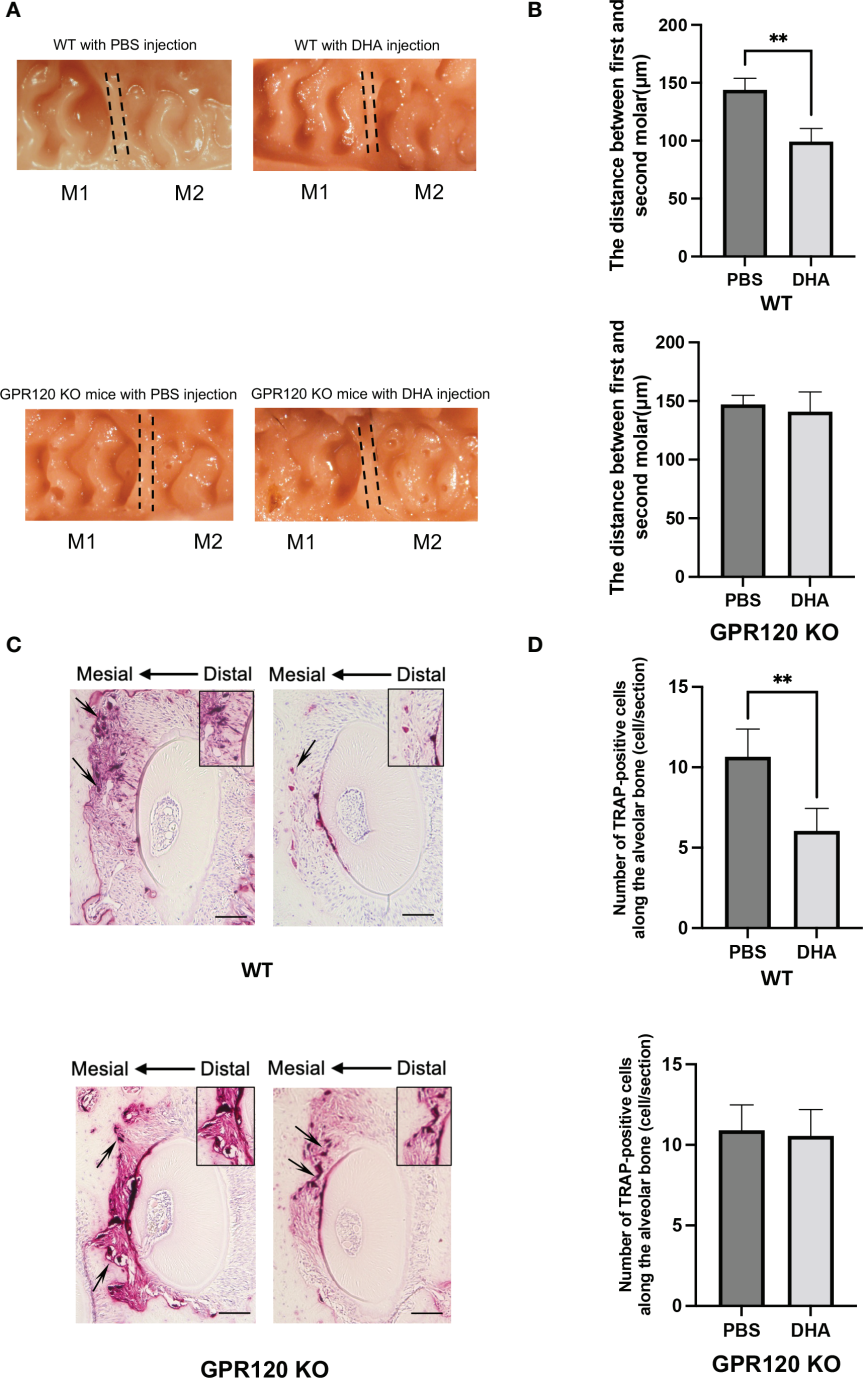
Tooth movement in WT and GPR120-KO mice treated with or without DHA after 12 days of OTM. **(A)** Images of a 12-day duration of orthodontic tooth movement in WT and GPR120-KO mice with PBS or DHA. **(B)** Measurement of the distance between the first and the second molar in WT and GPR120-KO mice with PBS or DHA after 12-day force loading. **(C)** TRAP-stained histological sections of the distobuccal root of the maxillary left first molar of WT and GPR120-KO mice after a 12-day duration of orthodontic tooth movement. The arrows show the osteoclasts formation and bone resorption area along the root. **(D)** The number of TRAP-positive cells along the alveolar bone surface around the distobuccal root of the left maxillary first molar after 12-day orthodontic tooth movement of WT and GPR120-KO mice treated with PBS or DHA. The results are given as means ± SD. Scheffé’s test was used to determine the statistical significance of differences (n = 4; **p < 0.01). Scale bars = 50 μm.

A 12-day OTM was examined in WT and GPR120-KO mice treated with PBS or DHA. The OTM distance on day 12 was significantly different between control (PBS) and DHA-treated WT mice (143.9 ± 10.0 μm and 99.2 ± 11.4 μm, respectively) (**Figures 6A, B**). In contrast, the OTM distance in control (PBS) and DHA-treated GPR120-KO mice were at the same level (147.0 ± 7.8 μm and 141.0 ± 16.8 μm, respectively) (**Figures 6A, B**).

Sections of five different levels from the distobuccal root of the upper left first molar were subjected to TRAP staining. A large number of TRAP-positive cells were noticed along the alveolar bone in the compression side of the tooth in control WT mice on day 12, but DHA-treated WT mice showed fewer TRAP-positive cells (**Figures 6C, D**). However, the number of TRAP-positive cells was similar in DHA-treated GPR120-KO mice and the control group on day 12 (**Figures 6C, D**).

## Discussion

DHA has been reported to have several positive impacts on individual health, including anti-inflammatory effects. DHA was reported to inhibit osteoclast formation *via* the omega-3 fatty acid receptor, GPR120. Orthodontic force induces TNF-α, which activates osteoclast differentiation during OTM. In this study, we investigated the impact of DHA on osteoclast formation, bone resorption induced by TNF-α, and OTM *in vivo*. Our results indicated that DHA attenuated TNF-α-induced osteoclast formation and bone resorption in WT mice but had no noticeable effect in GPR120-KO mice *in vivo*, suggesting that the attenuation of TNF-α-induced osteoclast bone resorption by DHA is mediated through GPR120. We previously reported that DHA directly inhibits osteoclast formation *via* GPR120 in osteoclast precursors. However, the effect of DHA on the expression of RANKL induced by TNF-α in osteoblasts is yet to be elucidated. Therefore, in the present study, we evaluated whether DHA inhibited TNF-α-induced RANKL expression in osteoblasts. The results showed that DHA inhibited TNF-α-induced RANKL expression in osteoblasts. Furthermore, to examine whether DHA inhibited TNF-α-induced RANKL expression in osteoblasts can indirectly inhibit osteoclast formation, we used GPR120-KO osteoclast precursors to avoid the direct effects of DHA and GPR120-KO osteoblasts to eliminate the effects of DHA. Our data demonstrated that DHA inhibited TNF-α-induced osteoclast formation in a co-culture system *via* activation of GPR120 in osteoblasts. As TNF-α plays a crucial role in OTM, DHA may inhibit TNF-α-induced osteoclast formation and bone resorption during OTM. Therefore, we performed OTM experiments in both WT and GPR120-KO mice with PBS or DHA treatment, which indicated that DHA also suppressed OTM in WT but not in GPR120-KO mice.

DHA was reported to prevent bone loss in ovariectomized mice by inhibiting the production and activation of osteoclasts *in vivo* (28). Moreover, experiments in rats have shown that perinatal supplementation with DHA reduced bone resorption, decreased osteoclast density, and increased bone mass (49). Treatment of oral *Porphyromonas gingivalis* infection-induced periodontitis model rats with fish oil containing DHA was reported to result in a marked reduction in alveolar bone resorption (50). Our previous study has suggested that DHA inhibited osteoclastogenesis and bone destruction induced by LPS *in vivo*, and this effect was ameliorated by the selective GPR120 antagonist, AH7614, in the LPS and DHA treatment group, indicating that DHA prevents LPS-induced osteoclastogenesis through GPR120 *in vivo* (31). Furthermore, TNF-α induces osteoclast formation both *in vivo* and *in vitro* (11–15), and we previously have reported that DHA inhibited TNF-α-induced osteoclast formation *in vitro* (31). However, the effects of DHA on TNF-α-induced osteoclast formation and bone resorption *in vivo* were not clear. Therefore, this study was conducted to examine whether DHA inhibited TNF-α-induced osteoclast formation. Daily injection of 100 μg of DHA to the supracalvaria for 5 days suppressed osteoclast formation induced by TNF-α. The impact of DHA on TNF-α-induced bone destruction was examined based on the ratio of bone destruction area to total area on micro-CT images. Consistent with previous studies, the TNF-α and DHA administration group showed significantly reduced bone resorption compared to the group administered TNF-α alone, indicating that DHA suppressed osteoclast formation and bone destruction *in vivo* (29–31, 51, 52). Using GPR120-KO mice, we also examined whether the suppression of osteoclastogenesis and bone destruction induced by TNF-α *via* DHA *in vivo* was mediated through GPR120. Although TNF-α-induced osteoclastogenesis and bone destruction suppressed by DHA *in vivo* was detected in WT mice, the effects were not observed in GPR120-KO mice. Taken together, the suppression effect of DHA on osteoclastogenesis and bone resorption mediated by TNF-α *in vivo* is mediated through GPR120.

DHA was concluded to suppress the production of inflammatory cytokines by several types of cells. DHA was shown to prevent IL-6 and TNF-α expression in primary mouse macrophages and the mouse macrophage cell line RAW247.6 by binding to GPR120 (23). DHA inhibits the inflammatory cytokines, such as IL-6, and TNF-α and IL-1β expression induced by LPS in THP-1 cells, which is the human monocytic leukemia cell line (53). Additionally, DHA downregulates the production of IL-1β in bone-marrow-derived macrophages (54). Our previous study showed that the TNF-α mRNA expression level A in macrophages and RANKL mRNA in stromal cells were reduced in mice treated with DHA and LPS compared to those treated with LPS alone (31). There are two possible mechanisms by which DHA suppresses TNF-α-induced osteoclastogenesis and bone resorption *in vivo*. First, previous studies have shown that DHA may directly inhibit TNF-α-induced osteoclastogenesis by affecting cell differentiation *in vivo*. Our previous study has shown that DHA directly inhibits TNF-α-induced differentiation of osteoclast progenitors into osteoclasts *in vitro* (31). Second, DHA may inhibit the TNF-α-induced expression of RANKL, which promotes osteoclast formation, in osteoblasts *in vivo*. RANKL and TNF-α are known to be essential factors in osteoclast formation (10–13), and several studies have shown that TNF-α induces the expression of RANKL in stromal cells, including osteoblasts and osteocytes (55–62). In this study, RANKL and Cathepsin K mRNA expression increased in mice treated with TNF-α but decreased in mice treated with a combination of DHA and TNF-α. These results correspond with the hypothesis that DHA suppresses osteoclast formation by inhibiting TNF-α-induced RANKL expression. We also examined whether DHA suppresses TNF-α-induced RANKL and Cathepsin K mRNA expression *via* GPR120 *in vivo* through experiments using GPR120-KO mice. Our results showed that TNF-α-induced RANKL and Cathepsin K mRNA expression was not inhibited by DHA in GPR120-KO mice, indicating that DHA inhibits TNF-α-induced RANKL and Cathepsin K production *via* GPR120 *in vivo*. Taken together, our observations suggest that the inhibitory effect of DHA on TNF-α-induced osteoclast formation *in vivo* may be due to both reduction in RANKL, Cathepsin K expression, and RANKL/OPG ratio, and a direct effect of DHA on TNF-α-induced osteoclast formation *via* GPR120 in osteoclast precursors as well.

This study showed that DHA inhibited TNF-α-induced RANKL expression in osteoblasts. As our previous study has shown that DHA directly inhibited TNF-α-induced osteoclast formation (31), we examined whether the inhibition of TNF-α-induced osteoclast formation by DHA was mediated *via* inhibition of RANKL expression in osteoblasts in a co-culture system using GPR120-KO osteoclast precursors to eliminate the direct effects of DHA on osteoclast precursors and WT osteoblasts. TNF-α was shown to enhance osteoclast formation; however, DHA inhibited TNF-α-enhanced osteoclast formation in co-cultures of GPR120-KO osteoclast precursors and WT osteoblasts. Furthermore, we examined whether DHA inhibited TNF-α-induced osteoclast formation *via* GPR120 in a co-culture system using GPR120-KO osteoclast precursors and GPR120-KO osteoblasts. No inhibition of osteoclast formation was observed in this co-culture system, suggesting that DHA acts on osteoblasts to decrease RANKL expression induced by TNF-α *via* activation of GPR120 in osteoblasts.

NF-κB is an important mediator in the process of inflammation, which facilitates intracellular signaling of inflammatory cytokines. Dysregulated NF-κB activation is a sign of chronic inflammatory diseases (63). The p50/p65 dimer of the NF-κB subunits is found in the cytoplasm, where its activation is repressed by IκBα. Upon stimulation in inflammatory conditions such as in rheumatoid arthritis by inflammatory cytokines, the IκB kinase (IKK) complex phosphorylates IκBα for subsequent ubiquitination and proteasome-mediated degradation, and IκBα dissociation induces NF-κB p65 subunit localization to the nucleus and binding to specific gene promoters to regulate pro- and anti-inflammatory protein expression. In this study, we examined the nuclear localization of NF-κB P65 subunit to figure out the effects of DHA on NF-κB signaling in WT and GPR120 KO mice. The results demonstrated that the nuclear localization of P65 was obviously increased in the TNF-α-stimulated group of both WT and GPR120-KO mice. Furthermore, the ratio of NF-κB activated cell was significantly decreased in WT DHA and TNF-α co-administrated group. However, the NF-κB-activated cell remained at the same level in the co-administrated group as TNF-α only group in GPR120-KO mice. These results indicate that DHA inhibits NF-κB activation induced by TNF-α in osteoblast through GPR120.

Several reports have shown that the compressive force in OTM induces the expression of TNF-α, which plays a significant role in osteoclast formation (64–69). Our previous study has also shown that TNF-α plays a crucial role in OTM in mouse models, where the distance of tooth movement was shorter in TNF-receptor-deficient mice than WT mice (41). These results indicated that TNF-α has a significant influence on OTM and therefore suggested that DHA may inhibit TNF-α-induced osteoclast formation during OTM. Regular injection of DHA was shown to inhibit OTM and osteoclast formation during OTM in WT, but not GPR120-KO mice, indicating that the effects of DHA on OTM and osteoclast formation during OTM are mediated *via* GPR120. Previous studies using chimeric TNF-receptor-deficient mice and WT mice have shown that stromal cells made a greater contribution to TNF-α-induced osteoclast formation than macrophages *in vivo* (14, 15). These studies concluded that TNF-α-responsive stromal cells contributed more to TNF-α-induced osteoclast formation than macrophages did *in vivo*. In another study, we also used chimeric TNF-receptor-deficient mice and WT mice to examine the contributions of each cell type *in vivo* during OTM, and the results indicated that the osteoclast number and distance of OTM were higher in chimeric mice with TNF receptors on stromal cells (70). These findings showed that the TNF-α responsiveness of stromal cells is an essential factor for osteoclastogenesis and bone destruction in OTM. In our present study, DHA was shown to inhibit TNF-α-induced RANKL expression and osteoclast formation *in vivo*. Additionally, DHA inhibited RANKL expression in osteoblasts *via* TNF-α *in vitro*. Furthermore, DHA inhibited OTM and osteoclast formation during OTM. These results suggested that DHA may inhibit TNF-α-responsive osteoblasts in OTM. The results of the present study taken together with those of previous studies suggest that DHA may inhibit TNF-α expression, TNF-α-induced osteoclast formation, and RANKL expression in TNF-α-responsive osteoblasts *via* GPR120 in OTM.

## Conclusions

**Figure 7:**
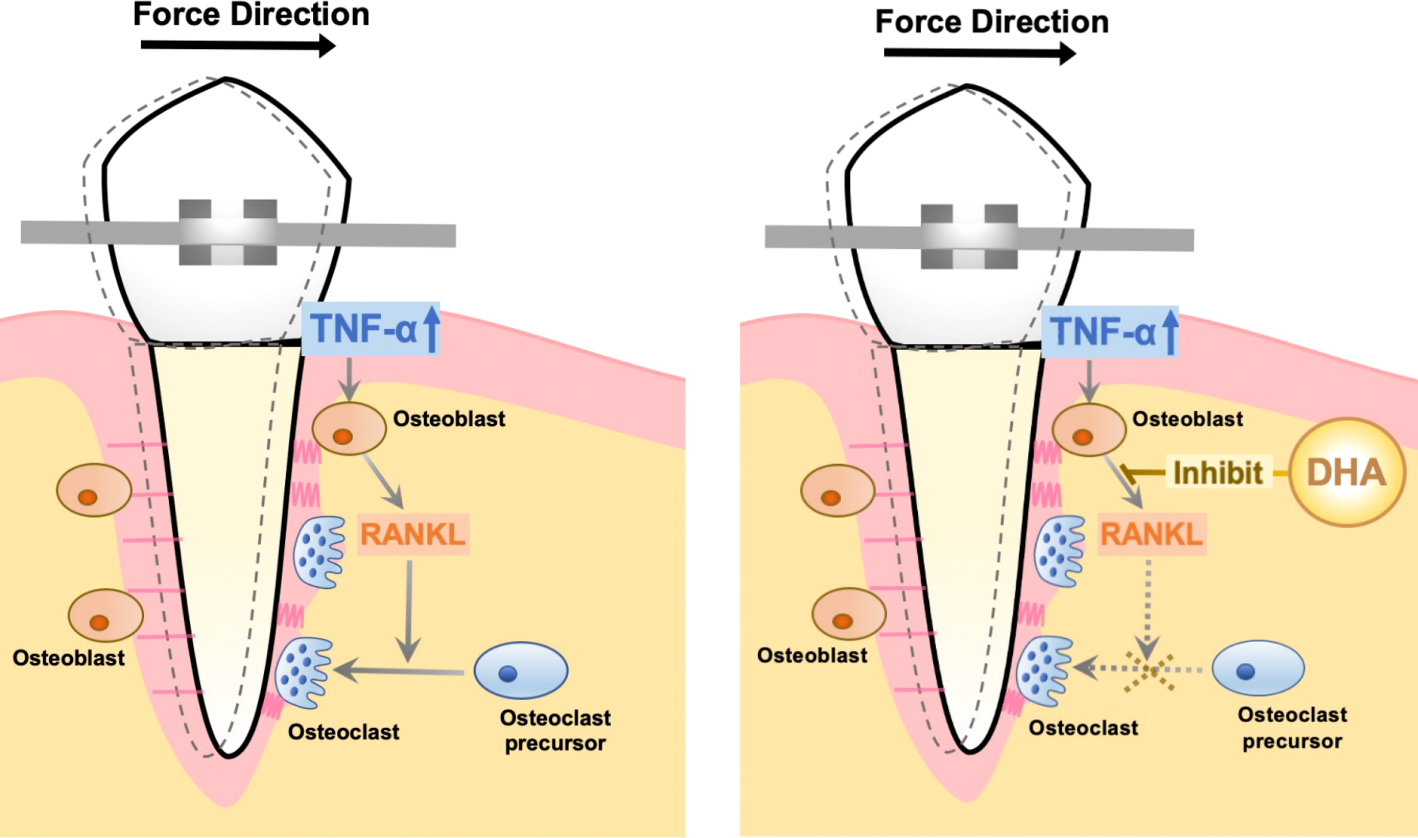
Schema of the mechanism of DHA-inhibited osteoclast formation during OTM.

In this study, we concluded that DHA suppresses bone resorption induced by TNF-α *in vivo via* GPR120, and the same tendency was also demonstrated in OTM. Additionally, DHA directly restrains RANKL expression in osteoblasts *in vitro via* GPR120. The mechanisms by which DHA inhibits osteoclast formation and bone destruction induced by TNF-α *in vivo* were associated with its inhibitory effect on TNF-α-induced RANKL expression in osteoblasts *via* GPR120 (**Figure 7**).

## Data availability statement

The original contributions presented in the study are included in the article/supplementary material, further inquiries can be directed to the corresponding author.

## Ethics statement

All animal care and experiments were approved by the Tohoku University of Science Animal Care and Use Committee.

## Author contributions

Conceptualization, JM and HK. Methodology, HK. Validation, JM and HK. Formal analysis, JM, SO, and TN. Investigation, JM, HK, AM, FO, TN, AP, YN, RK, AK, and KK. Resources, HK. Data curation, JM. Writing (original draft preparation), JM. Writing (review and editing), HK. Supervision, AI and IM. Project administration, HK. Funding acquisition, AK, HK, and IM. All authors contributed to the article and approved the submitted version.
